# Associations of Brain Arteriovenous Malformation-Related Factors with Epileptic Seizure Presentations

**DOI:** 10.3390/diagnostics14111077

**Published:** 2024-05-22

**Authors:** Kymbat Mukhtarova, Chingiz Nurimanov, Elena Zholdybayeva, Yerbol Makhambetov, Serik Akshulakov

**Affiliations:** 1 National Center for Biotechnology, 13/5, Kurgalzhynskoye Road, Astana 010000, Kazakhstan; kymbat.mukhtarova@alumni.nu.edu.kz; 2 Department of Vascular and Functional Neurosurgery, National Center for Neurosurgery, 34/1 Turan Avenue, Astana 010000, Kazakhstanyermakh@gmail.com (Y.M.); raim@rambler.ru (S.A.)

**Keywords:** brain arteriovenous malformations, epilepsy seizures

## Abstract

Background: Arteriovenous malformations (AVMs) are abnormal tangles of arteries and veins that connect directly without an intervening capillary bed. Epileptic seizures are the second most common symptom in patients with brain AVMs, occurring in 30 to 50% of cases. However, the exact mechanism of epileptic seizure development in AVMs remains unclear. In this study, we aimed to investigate the factors associated with epileptic seizures in patients with brain arteriovenous malformation (AVMs) in Kazakhstan. Methods: A case–control study was conducted, which included 163 patients diagnosed with brain AVMs. Demographic and clinical data were collected and analyzed, and multivariate logistic regression was built to assess the factors associated with seizures in brain AVMs. Results: from this rupture of vessels OR = 0.36 95% CI (0.14–0.91, a medium-to-high Spetzler-Martin score (III–V) OR = 6.16 (2.14–17.69) and OR = 3.05 (1.08–8.68), respectively), location in brain cortex (frontal lobe OR = 6.16 (2.04–18.54), parietal lobe OR = 9.37 (3.26–26.91), temporal lobe OR = 4.57 (1.56–13.36), occipital lobe OR = 0.27 (0.08–0.91), and the presence of hemiparesis OR = 0.12 (0.02–0.66) in adverse outcomes were statistically significantly associated with the presence of epileptic seizures in brain arteriovenous malformations patients. Conclusions: To conclude, this contributed to model factors associated with brain arteriovenous malformations that are linked to epileptic seizures.

## 1. Introduction

Arteriovenous malformations (AVMs) are abnormal tangles of arteries and veins that directly connect without an intervening capillary bed. This can lead to the dilation and rupture of blood vessels. The main goal of the interventions for AVMs is to reduce or eliminate the risk of intracranial hemorrhage in AVM patients [1], which ranges from 37% to 71% [2].

Seizure is the second most common symptom of AVMs. However, the exact mechanism behind the development of epileptic seizures in AVMs remains unclear. In a review by M. Bustuchina Vlaicu, the findings of recent studies on epilepsy associated with brain AVMs were summarized. The author highlights that the development of brain AVMs is attributed to multiple factors. The review also highlights the lack of sufficient published data to determine the most suitable treatment for these patients [3].

Nevertheless, several authors have reported associations with various factors, including size, superficial vein drainage, location in the cortex, feeding by the middle cerebral artery, absence of aneurysms, presence of varix/varices in the venous drainage, male sex, frontal lobe, and arterial border-zone location [4,5,6,7,8,9]. In addition, Hoh and colleagues [10] reported associations with male sex, age of less than 65 years, AVM size of more than 3 cm, and temporal lobe location in patients who experienced epileptic seizures after undergoing surgeries for AVMs [11]. The prevalence of epileptic seizures in AVM patients is estimated to be 30% [1]. Epileptic seizures are present in 30 to 50% of AVM patients [10]. Different treatment options such as surgical resection, radiosurgery, or embolization procedures can be used. However, more studies are needed in different countries to better understand epileptogenesis and gather clinical data and characteristics of patients with brain AVM-associated epileptic seizures. This study aims to identify characteristics of AVMs that could be associated with the clinical presentation of epilepsy in a sample of patients from Kazakhstan.

## 2. Methods

### 2.1. Study Design

A case–control study was conducted to determine the factors associated with epilepsy in brain AVM patients in Kazakhstan. The inclusion criteria required participants to be diagnosed with brain arteriovenous malformations confirmed through MRI and cerebral angiography scans and to be older than 18. There were no exclusions based on gender or ethnicity. The cases consisted of patients diagnosed with epilepsy, while controls were brain AVMs subjects without any signs of epilepsy. Both clinical and instrumental characteristics, such as EEG, were analyzed and classified by the neurologist according to the International League Against Epilepsy (ILAE) and Engel classification. In addition, the patients were prescribed anti-epileptic drugs (AED) by epileptologists to manage their seizures. In total, 163 eligible patients were recruited from the National Center for Neurosurgery in Astana, Kazakhstan. The clinical records of these patients were collected between June 2020 and August 2022. Ethical approval for the study was obtained from the National Center for Biotechnology (#2/1 August 2019, Astana, Kazakhstan). All study participants provided written informed consent forms. To protect their identity and minimize potential risks associated with participation in this study, all personal information about the participants was de-identified.

### 2.2. Variables

Clinical information for this study was collected using basic demographic data, and characteristics of AVMs, including clinical symptoms. The independent variables included age, sex, weight, and height at diagnosis. Body mass index (BMI) was then calculated following the guidelines. The clinical symptoms used in this study were the presence or absence of cephalgia, hemiparesis, and seizures. The previous medical history was also considered, including arterial hypertension, smoking status, self-reported family history of AVM, and intracranial hemorrhage in relatives. AVM characteristics that were examined included location (cortex, cerebellum, brain stem, or basal ganglia), venous drainage (deep or superficial), size of the nidus (0–3 cm, 3–6 cm, or larger than 6 cm), presence of aneurysms, and Spetzler-Martin scores. The dependent variable of interest was the presence or absence of epileptic seizures associated with brain AVM. The Spetzler-Martin Grade, a system proposed in 1986 to assess the risk of open neurosurgery for AVM patients, was used. This grading system considers the size of the AVM, patterns of venous drainage, and eloquence of the adjacent brain. Grades I and II are typically removed without much difficulty in a single-step resection, while Grades IV and V usually require embolization and several rounds of resection [12]. Grade III AVMs typically require a multimodal approach involving embolization, radiosurgery, and resection. Grade IV and V AVMs are usually monitored if they have not ruptured [3]. Therefore, the risk of surgical intervention should not outweigh the potential risks associated with leaving the AVM unresected. Based on this information, the Spetzler-Martin grades were grouped into three categories for further statistical analysis: low grade (I and II), medium grade (III), and high grade (IV–V).

### 2.3. Statistical Analysis

Statistical analysis was conducted using STATA 14.2 software (StataCorp, College Station, TX, USA), and data management was performed in Microsoft Excel, version 2404 (Microsoft Office (Microsoft Corp., Redmond, WA, USA)). Descriptive statistics were generated, and the association between hemorrhage and the variables was examined. Fisher’s exact test was used for categorical independent variables, while the Wilcoxon Rank Sum test was used for continuous variables. A *p*-value of 0.2 was set as the cutoff for univariate logistic regression to determine which variables would be considered for the multivariate logistic regression (MLR) model building. Only statistically significant covariates were included in the MLR model. All statistical tests were two-sided, and a significance level (α) of 5% was used for all tests.

## 3. Results

### 3.1. Study Subjects

**Figure 1:**
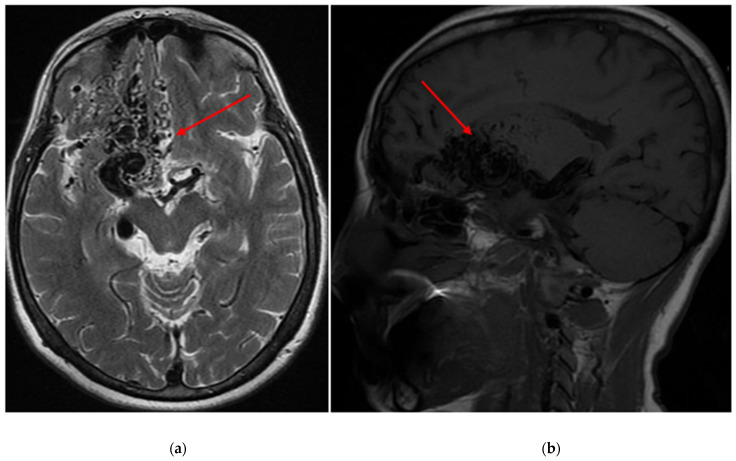
(**a**) An axial T2 magnetic resonance image of the brain depicts an arteriovenous malformation (AVM) situated within the right frontal lobe (red arrows), with a Spetzler-Martin grade of IV assigned to it (**b**) A sagittal T1 magnetic resonance image of the brain reveals the same AVM located within the right frontal lobe, also assigned a Spetzler-Martin grade of IV.

**Figure 2:**
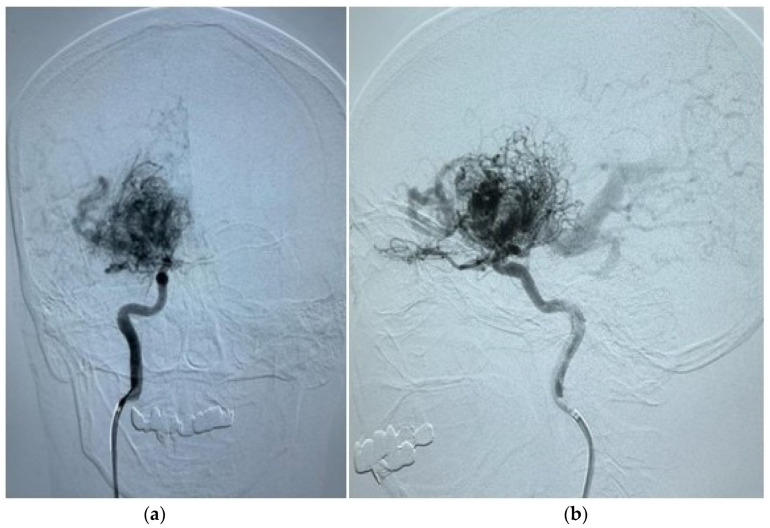
(**a**) A cerebral angiography image in the antero-post position displays an afferent artery originating from both the right middle cerebral artery and the right internal carotid artery, supplying the AVM. Additionally, it reveals an extended deep draining vein. (**b**) Another cerebral angiography image in the lateral position exhibits identical afferent arteries and enlarged draining veins associated with the AVM within the right frontal lobe.

Demographic and clinical data of AVM sampling are shown in Table 1. Seventy patients presented with AVM-associated seizures, and 93 participants had no signs of seizures. Figure 1 and Figure 2 show magnetic resonance imaging (MRI) and cerebral angiography images of the brain of a patient with seizures associated with arteriovenous malformations. In total, 55.71% of subjects with seizures and 56.99% of AVMs without seizures were female. The age and BMI composition of cases and controls were comparable and not statistically significantly different (*p* > 0.05). Almost 95% of all participants underwent partial or total embolization (Table 2). The most common symptom was rupture, present in 41.98% of all participants.

### 3.2. Association Study

Demographic variables do not appear to affect seizure probability in this sample of patients. Table 1 and Table 2 present the baseline characteristics. The following factors were found to be statistically significant in epilepsy versus no epilepsy associated with brain AVM: the size of the nidus (*p* = 0.000), rupture of AVM (*p* = 0.000), location in the cortex (which includes frontal, parietal, or temporal lobes) (*p* = 0.000), location in the cerebellum, and hemiparesis in complications (*p* = 0.012). Aneurysms were equally present in both groups (*p* = 1.000).

The multivariate regression model included rupture of the AVM, location in the cortex (frontal, temporal, parietal, and occipital lobes), hemiparesis, and Spetzler-Martin grade. The model was found to be statistically significant overall (*p* < 0.000), and the R2 was approximately 32.3%.

Importantly, the rupture of AVM was found to be protective, with an odds ratio (OR) of 0.36 and a 95% confidence interval (CI) of 0.14–0.91. In other words, a ruptured AVM was associated with an approximately 64% lower chance of epilepsy in adverse outcomes, with a 95% confidence interval ranging from 86% to 9% lower chance of epilepsy. However, Spetzler-Martin grade III was associated with a higher chance of epilepsy in adverse outcomes. Specifically, there is a 95% confidence that the true effect of Spetzler-Martin grade III lies within a range of 2.14 to 17.69. Additionally, location in the brain cortex is associated with higher odds of epilepsy in brain AVM patients. Specifically, location in the frontal lobe is associated with 2.04 to 18.54 times odds (*p* = 0.001); parietal lobe 3.26–26.91 times odds (*p* = 0.000); temporal lobe 1.56–13.36 times odds (*p* = 0.005); occipital lobe 0.08 to 0.91 times odds (*p* = 0.035) of epileptic seizures in AVM patients. Hemiparesis is associated with an OR of 0.12, with a 95% CI ranging from 0.02–0.66 chance of seizures in AVM subjects (Table 3).

## 4. Discussion

Factors associated with epilepsy at initial presentation were identified from our cohort of 163 brain arteriovenous malformation patients. These factors include hemiparesis, Spetzler-Martin score above III, frontal, parietal, occipital, or temporal lobe location, and rupture.

Spetzler-Martin grading is used to assess the risk of AVM and determine the appropriate management strategy. In the case of low-grade AVMs (I and II) which have a low risk of mortality and morbidity, treatment options are usually successful and offered in most cases. On the other hand, high-grade AVMs (IV and V) are typically managed conservatively, with a risk versus benefit assessment conducted before any intervention. For example, Dicpinigaitis et al. [13] found that after conservative treatment, the frequency of hemorrhages increased threefold and mortality doubled. Capocci et al. suggested that exclusionary treatment in unresolved epilepsy patients is associated with a higher incidence of seizure absence compared to conservative treatment [14]. However, medium-grade AVMs (Spetzler-Martin score III) are often the subject of debate, because they fall between the two other grades [11]. According to our findings, Spetzler-Martin grade III demonstrated a statistically significant association with epileptic seizures in AVM subjects. Ching-Jen Chen et al. also demonstrated that the Spetzler-Martin score and the AVM radiological score in Virginia may help stratify the risk of seizures [15]. In the case of cortical AVMs, those located in the temporal lobe were found to be predictive of seizure presentation. Therefore, it may be important to consider epilepsy as a significant factor in decision-making, along with the risk of intracranial hemorrhage.

Interestingly, rupture and hemiparesis were found to have a somewhat protective effect. This suggests that there may be a mechanism of epilepsy that is not directly associated with intracranial hemorrhage and ischemic stroke. One possible explanation for this is that the process of embolization, which involves blocking blood flow to the abnormal blood vessels of the AVM, could potentially reduce the occurrence of vascular steal syndrome and the mass effect caused by dilated AVM blood vessels. Vascular steal syndrome occurs when blood is diverted from normal brain tissue to the AVM, depriving the normal tissue of nutrients and oxygen. In this way, the brain tissue located near the AVM is susceptible to hypoperfusion [16]. According to Bokhari and Bokhari [17], seizures can occur as a result of the mass effect of the AVM or venous hypertension in the draining veins, even in the absence of hemorrhage.

Rajeev In Sen studied de novo epilepsy following microsurgical resection of brain arteriovenous malformations. In cases where de novo epilepsy arises after AVM resection, the risk of annual cumulative occurrence is 9%, with the potential for long-term onset. Poorly controlled epilepsy may be associated with the location of the temporal lobe and the time lapse between hemorrhage and resection [18].

Thus, this paves the way for future investigations aimed at enlarging the sample size to enhance the robustness of findings, providing precise delineation of seizure manifestations, and incorporating comprehensive data regarding the treatment protocols administered to individuals with AVMs.

There are several limitations to the study. Firstly, the selection of patients was not random. Each AVM characteristic requires specialized intervention based on the potential risks and benefits for the patients, which can affect the outcome. Secondly, the sample size is relatively small, which may lead to unreliable results. Thirdly, patients with prior embolizations and other interventions were not excluded. This could introduce bias, as there is existing evidence of the modifying effect of interventions on epilepsy and seizures.

## 5. Conclusions

To summarize, this study found that unruptured AVMs with medium-to-high Spetzler-Martin scores, located in the frontal, parietal, or temporal lobes, pose a higher risk of epileptic seizures in patients without hemiparesis.

## Figures and Tables

**Table 1:** Demographic and clinical characteristics.

Variables	Total (*n* = 163)		Seizures (*n* = 70)		No Seizures (*n* = 93)		*p*-Value
Age	**39.21 ± 12.75**		**39.91 ± 11.49**		38.63 ± 13.69		0.397
Gender							0.875
male	71	43.56%	31	44.29%	40	43.01%	
female	92	56.44%	39	55.71%	53	56.99%	
Height	167.44 ± 9.11		168.03 ± 8.91		166.97 ± 9.32		0.489
Weight	68.9 ± 14.42		69.84 ± 14.23		68.15 ± 14.68		0.161
BMI	24.68 ± 4.74		24.97 ± 4.35		24.45 ± 5.05		0.248
Arterial hypertension	32	19.63%	11	15.71%	21	22.58%	0.322
Smoke	44	26.99%	21	30%	23	24.73%	0.480
Intracranial hemorrhage in relatives	40	32.79%	16	32%	24	33.33%	1.000
Rupture	68	41.98%	18	26.09%	50	53.76%	0.000 **
AVM relatives	3	2.38%	0	0.00%	3	4.11%	0.263
Aneurysm	15	9.2%	6	8.57%	9	9.68%	1.000
Cephalgic syndrome	17	10.49%	5	7.25%	12	12.9%	0.305
Hemiparesis	22	13.58%	3	4.35%	19	20.43%	0.004 *

* *p* < 0.01, ** *p* < 0.001.

**Table 2:** AVM characteristics.

Variables	Total (*n* = 163)		Seizures (*n* = 70)		No Seizures (*n* = 93)		*p*-Value
**Size of AVM**							0.000 ***
0–3	47	29.01%	11	15.71%	36	39.13%	
3–6	97	59.88%	54	77.14%	43	46.74%	
6+	18	11.11%	5	7.14%	13	14.13%	
**Location in cortex**	134	83.75%	68	97.14%	66	73.33%	0.000 ***
Frontal lobe	45	28.13%	30	42.86%	15	16.67%	0.000 ***
Parietal lobe	62	38.75%	37	52.86%	25	27.78%	0.002 **
Temporal lobe	49	30.63%	27	38.57%	22	24.44%	0.060
Occipital lobe	31	19.38%	7	10%	24	26.67%	0.009 **
**Cerebellum**	12	7.5%	1	1.43%	11	12.22%	0.013 *
**Brain stem**	4	2.5%	0	0.0%	4	4.44%	0.132
**Basal ganglia**	24	15%	7	10%	17	18.89%	0.180
**Vein drainage**							0.411
deep	71	47.65%	27	43.55%	44	50.57%	
surface	78	52.35%	35	56.45%	43	49.43%	
**Spetzler-Martin Score**							0.029 *
Low (I–II)	47	29.19%	13	18.57%	34	37.36%	
Medium (III)	55	34.16%	29	41.43%	26	28.57%	
High (IV–V)	59	36.65%	28	40.00%	31	34.07%	
**Location**							0.869
Left	88	58.28%	40	57.14%	48	59.26%	
Right	63	41.72%	30	42.86%	33	40.74%	
**Prior embolization**	143	94.08%	67	98.53%	76	90.48%	0.043 *
**Prior SRS**	14	9.21%	4	5.88%	10	11.90%	0.264
**Prior craniotomy**	12	7.89%	6	8.82%	6	7.14%	0.768

* *p* < 0.05, ** *p* < 0.01, *** *p* < 0.001.

**Table 3:** Multivariate regression model for factors associated with brain AVM-associated epilepsy.

Variable	OR (95% CI)	*p*-Value
Ruptured		
yes	0.36 (0.14–0.91)	0.032 *
no	Reference	
Spetzler-Martin score		
Low (I–II)	Reference	
Medium (III)	6.16 (2.14–17.69)	0.001 ***
High (IV–V)	3.05 (1.08–8.68)	0.036 *
Frontal Lobe	6.16 (2.04–18.54)	0.001 ***
Parietal Lobe	9.37 (3.26–26.91)	0.000 ***
Temporal Lobe	4.57 (1.56–13.36)	0.005 **
Occipital Lobe	0.27 (0.08–0.91)	0.035 *
Hemiparesis		
yes	0.12 (0.02–0.66)	0.015 *
no	Reference	

* *p* < 0.05, ** *p* < 0.01, *** *p* < 0.001; reference denotes OR = 1.

## Data Availability

Data can be provided upon request.

## Abbreviations

AVM, arteriovenous malformation; MRI, magnetic resonance imaging; BMI, Body mass index; MLR, multivariate logistic regression; SRS, stereotactic radiosurgery.
